# MicroRNA-221 Modulates Airway Remodeling via the PI3K/AKT Pathway in OVA-Induced Chronic Murine Asthma

**DOI:** 10.3389/fcell.2020.00495

**Published:** 2020-06-30

**Authors:** Jing Pan, Qianyuan Yang, Yao Zhou, Huan Deng, Yifan Zhu, Deyu Zhao, Feng Liu

**Affiliations:** ^1^Department of Respiratory Medicine, Children’s Hospital of Nanjing Medical University, Nanjing, China; ^2^Department of Emergency/Critical Medicine, Children’s Hospital of Nanjing Medical University, Nanjing, China; ^3^Department of Pediatrics, The Affiliated Changzhou No. 2 People’s Hospital of Nanjing Medical University, Changzhou, China

**Keywords:** miR-221, asthma, airway inflammation, airway remodeling, ASMCs

## Abstract

**Background:**

Airway remodeling is one of the most important pathological features of chronic asthma. This study aimed to determine whether microRNA-221 (hereafter, miR-221) can affect airway remodeling in a mouse model of ovalbumin (OVA)-induced chronic asthma.

**Methods:**

Adeno-associated viruses (AAVs) “Bearing miR-221 sponges” were used to downregulate miR-221 in asthmatic mice. Staining with hematoxylin and eosin (H&E), Masson trichrome, and periodic acid–Schiff reagent was used to assess histological changes. The affected signaling pathway in mouse airway smooth muscle cells (ASMCs) was also identified by gene chip technology. A PI3K/AKT-inhibitor (LY294002) was used to confirm the role of the pathway in ASMCs.

**Results:**

The inhibition of miR-221 in a murine asthma model was found to reduce airway hyper-responsiveness, mucus metaplasia, airway inflammation, and airway remodeling (*p* < 0.05). Furthermore, miR-221 was found to regulate collagen deposition in the extracellular matrix (ECM) of ASMCs. Bioinformatics analysis and western blot analysis confirmed that the PI3K-AKT pathway was involved in ECM deposition in ASMCs.

**Conclusion:**

miR-221 might play a crucial role in the mechanism of remodeling via the PI3K/AKT pathway in chronic asthma and it could be considered as a potential target for developing therapeutic strategies.

## Introduction

Asthma is a chronic pulmonary inflammatory disease characterized by AHR and the inflammation and remodeling of airways. It is a major public health concern, with approximately 300 million people (especially children) being affected worldwide and over 30 million asthma patients in China alone (GINA) (Bateman et al., 2018). The direct costs and indirect expenses incurred as a result of asthma pose a considerable economic burden on families and society (Chen et al., 2018). Currently, corticosteroids are the most effective way to control asthma attacks (Veeranki et al., 2017). However, the efficacy of steroids is limited to airway remodeling traits such as basement membrane thickness and ECM deposition. Statistically, 5–25% of patients do not respond well to steroid therapy and are considered steroid-resistant (Hansbro et al., 2017).

The long-term recurrent stimulation of chronic inflammatory responses can result in the destruction of epithelial structure, which further leads to asthma airway remodeling (Boulet, 2018). Airway reconstruction is also considered the most important cause of irreversible air flow limitation and continuous AHR. The mechanisms regulating AHR and airway remodeling remain to be elucidated, necessitating the study of the pathogenesis of asthma at the molecular level (Russell and Brightling, 2017).

MicroRNAs are small non-coding RNAs, having important roles in the post-transcriptional regulation of gene expression. MiRNAs bind to the 3’-UTR of a target gene, resulting in translational repression and/or mRNA degradation (Shukla et al., 2011). MiRNAs also play a critical role in the regulation of innate immunity and inflammation in lung diseases such as asthma (Kai et al., 2015; Izabela et al., 2016). As an important effector of allergic diseases, miR-221 has been shown to regulate the cell cycle of mast cells (Mayoral et al., 2009). The contraction and relaxation function of smooth muscle cells is also regulated by miR-221 (Perry et al., 2014). Our previous studies found a significant increase in the expression level of miR-221 in the peripheral blood of children with asthma, and similar results were obtained in the lung tissue of a mouse asthma model (Qin et al., 2012).

To explore the effects of miR-221 *in vivo*, we constructed a mouse model displaying the basic features of chronic asthma and studied the effects of AAV intervention. Furthermore, to elucidate the underlying mechanisms, we examined the changes in signaling pathways in ECM deposition in mouse ASMCs stimulated by miR-221.

## Materials and Methods

### Animals

All animal experiments were approved by the local animal care committee of the National Defense Medical Center (approval number: IACUC-1702005). Six-week-old female BALB/c mice were purchased from the Animal Core Facility of Nanjing Medical University (Nanjing, China) and maintained under a specific pathogen-free environment at the Animal Center of Nanjing Medical University. Mice were housed in a light- and temperature-controlled room with free access to deionized drinking water and standard chow. Animals were acclimatized to the laboratory conditions for 1 week prior to the start of the experiments.

### Experimental Protocols

Sixty female BALB/c mice were randomly divided into four exposure groups: (1) the control group, (2) shRNA-miR-221 group, (3) OVA group, (4) and OVA + shRNA-miR-221 group. Mice were sensitized via intraperitoneal (i.p.) injection of 20 μg OVA (Grade V, Sigma-Aldrich, United States) absorbed onto 2 mg Imject Alum (Thermo, United States) in 200 μL sterilized 0.9% saline once a week from week 0 to week 2. Mice were then challenged through the respiratory tract with 5% aerosolized OVA in saline for 30 min thrice a week from week 3 to week 10. Mice those received 0.9% saline sensitization and a challenge of the same volume and frequency were used as controls. Twenty-four hours after the last challenge (day 72), mice were anesthetized for the subsequent experiments. BALF and lung tissues were processed for subsequent analysis. The detailed protocol of this study is shown in Supplementary Figure S2.

### Infection With AAVs

To accurately regulate lung exposure to AAVs at the beginning of the study (day 3), the OVA solutions were instilled intratracheally to the mice. Such a practice greatly alleviated the pain of the mice and satisfied the experimental requirements. Mice were anesthetized by intraperitoneal injection of 4% chloral hydrate. Then, each mouse was suspended with the abdominal side facing outward on a nearly vertical incline by hooking its incisors on a rubber band at the top of the slope, with a light located above the mouse’s chest. The tongue of the mouse was gently pulled out with curved tweezers for the airway to be clearly visible by the light penetrating its chest. Then, the mice underwent endotracheal intubation with a venous indwelling needle (22G; BD, China). AAVs (50 μL, over 1 × 10^12^ vg/mL) were instilled into the lungs of mice through the venous indwelling needle inserted into the airway. The primer design sequences of miR-221-sponge were as follows: mmu-miR-221-3p-sponge-F: acaggatccGAAACCCAGCAGACAATGTAGCTtatacGAAACCC AGCAGACAATGTAGCTacatcGAAACCCAG; mmu-miR-221-3p-sponge-R: acagaattcAGCTACATTGTCTGCTGGGTTTC tgaagaAGCTACATTGTCTGCTGGGTTTCgatgtAGCTACA.

### Measurement of AHR

According to the manufacturer’s instructions of the AniRes2005 lung function system (Bestlab, version 2.0, China), 24 h after the final aerosol challenge, the mice were tested for AHR to methacholine (MeCh; Sigma-Aldrich). Mice were anesthetized by intraperitoneal injection of 1% pentobarbital sodium (Urchem, China). The respiratory rate was pre-set at 90/min, and the time ratio of expiration/inspiration was 20:10. AHR was assessed by the indexes of Re, Ri, and the minimum value of Cdyn (Cldyn). Ri and Re R-areas, the graph area between the peak value and baseline, and the valley of Cldyn were recorded for further analysis.

### BALF and Cell Counting

The trachea was exposed and intubated by a venous indwelling needle with a 1 mL sterile syringe filter. BAL was performed by the instillation of 0.5 mL saline through the trachea into the lung; the mouse’s chest was gently massaged, and the liquid was withdrawn. This process was repeated thrice. The total liquid recovered from different mice was ≥1.2 mL. Then, the BALF samples were centrifuged at 1,200 rpm at 4°C for 10 min. The cell sediment was suspended in saline to facilitate cell counting, placed on microscope slides, and stained with Wright–Giemsa stain. The percentage of eosinophils in the BALF was calculated by counting 100 cells on randomly selected areas of the slide using light microscopy (Olympus, Japan). Supernatants were harvested and stored at −80°C.

### Enzyme-Linked Immunosorbent Assay

The concentrations of IL-6 and TNF-α were measured in BALF and serum with the ELISA kit (Ray Biotech, United States). The assay was performed according to the manufacturer’s instructions.

### Lung Histological Assay

The left lungs of mice were harvested and fixed in 4% paraformaldehyde for 24 h and then embedded in paraffin using a standard protocol after dehydration. Then, 4 μm sections of embedded lung tissue were mounted onto slides and stained with H&E to identify tissue inflammation, with PAS to identify mucus production, and with Masson trichrome to evaluate collagen deposition and smooth muscle hyperplasia. Tissue sections were viewed with a microscope (Olympus) and Image Pro Plus 6.0 software.

### Cell Culture and Identification

Primary ASMCs were isolated from the tracheae of Sprague Dawley rats (Animal Core Facility, Nanjing Medical University) according to a previously reported method (Liu D et al., 2018). The culture medium was refreshed every 3 days with 10% FBS, and cells were used for subsequent experiments when they reached passages 6–10.

### RNA Isolation and RT-PCR

Total RNA was extracted from lung tissue and cultured ASMCs by TRIzol reagent (Invitrogen, Carlsbad, CA, United States) according to the manufacturer’s instructions. The cDNA was obtained using total RNA with a TaqMan MicroRNA Reverse Transcription Kit (Applied Biosystems, Waltham, MA, United States). The obtained cDNA was used to determine the expression of miR-221 in the lung tissues and ASMCs by qPCR in an Applied Biosystems 7500 Fast Real-Time PCR system (Thermo Fisher Scientific, Inc., Waltham, MA, United States) with a TaqMan MicroRNA Assay (Applied Biosystems; Thermo Fisher Scientific, Inc). The cDNA was also synthesized from total RNA by M-MLV reserve transcriptase (Takata, Dalian, China), and qPCR was performed to measure the expression of MUC5AC by SYBR Green PCR Master Mix (Applied Biosystems, Foster City, CA, United States) in the same system. Relative gene expression was calculated using the 2^–ΔΔCt^ method.

### Western Blot Analysis

Proteins were extracted from cultured cells or lung tissues using RIPA lysis buffer. Proteins were subsequently separated by electrophoresis using 10% sodium dodecyl sulfate polyacrylamide gel electrophoresis before being transferred to a PVDF membrane. Membranes were incubated with 5% skim milk for 2 h at 37°C before they were incubated with primary antibodies against COL3A1, COL1A2 (Bioworld Technology, Inc., St Louis Park, MN, United States), and GAPDH (Santa Cruz, Dallas, TX, United States) at 4°C overnight. The next day, the membranes were probed using horseradish peroxidase-conjugated secondary antibodies for 2 h at room temperature. Targeted protein signals were detected using Pierce ECL Western Blotting Substrate (Thermo Fisher Scientific, Inc.), and band intensities were measured using Image Pro Plus 6.0 software.

### Statistical Analysis

All data are presented as the mean ± standard error of the mean. Statistical graphs were generated using Graph-Pad Prism for Windows (ver. 6.00; GraphPad Software, San Diego, CA, United States). One-way ANOVA combined with Fisher’s protected *t*-test was used to determine the significance of the differences between groups. Differences at *p* < 0.05 and *p* < 0.01 were considered significant and extremely significant, respectively.

## Results

### Effects of miR-221 Inhibition on AHR

**FIGURE 1:**
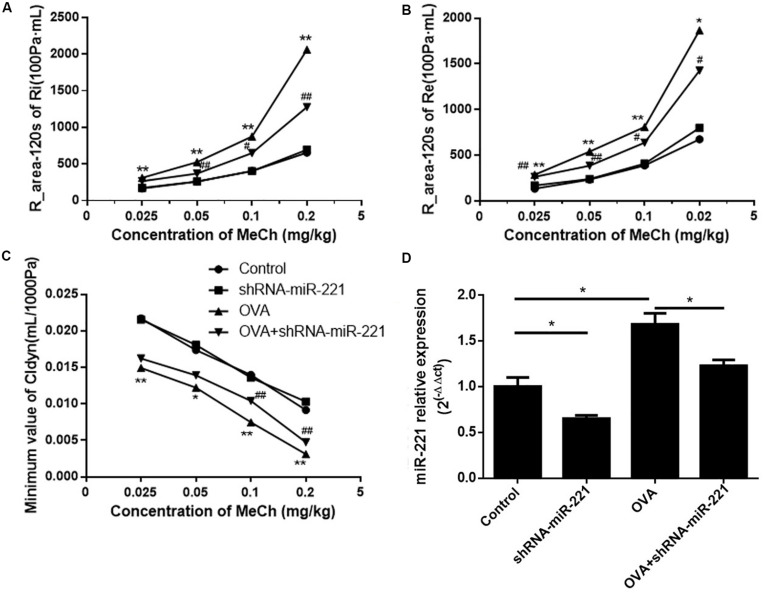
The expression of miR-221 in the lung tissues of mice with AAV infection and airway hyper-responsiveness measurements. **(A)** R-area of Re, **(B)** R-area of Ri, and **(C)** peak value of Cldyn at different doses of MeCh, **(D)** miR-221 expression in the lung tissues of mice with AAV infection. Animal groups (in all panels): *n* = 4 mice per group. *p < 0.05, **p < 0.01, compared with the saline control; ^#^*p* < 0.05, ^##^*p* < 0.01, compared with the OVA group.

To investigate the potential role of miR-221 in asthma, AAVs with miR-221-sponge were instilled intratracheally to the mice to downregulate miR-221. As shown in Figure 1D, the expression of miR-221 significantly increased in asthmatic mice compared with the control group and decreased in the OVA + shRNA-miR-221 group compared with the OVA group. In short, AAVs with miR-221-sponge could successfully inhibit the expression of miR-221 in the lung tissue of mice. To assess the changes in AHR, we compared airway responses to MeCh in different groups (Figures 1A–C). In all experimental groups, both the expiratory and inspiratory resistance increased with an increase in the MeCh dose, while the trough value of Cldyn decreased. At each point, OVA exposure had a significant influence on Ri, Re, and Cldyn (*p* < 0.05 or *p* < 0.01) between the saline group and OVA group. Compared with the OVA group, the inhibition of miR-221 could significantly reduce the changes in lung function (*p* < 0.05 or *p* < 0.01), mainly including reduced Ri and Re and increased dynamic lung compliance. This indicated that the inhibition of miR-221 could effectively reduce the changes in lung function induced by chronic asthma.

### Effects of miR-221 Inhibition on Inflammatory Cell Recruitment and the Level of Cytokines

**FIGURE 2:**
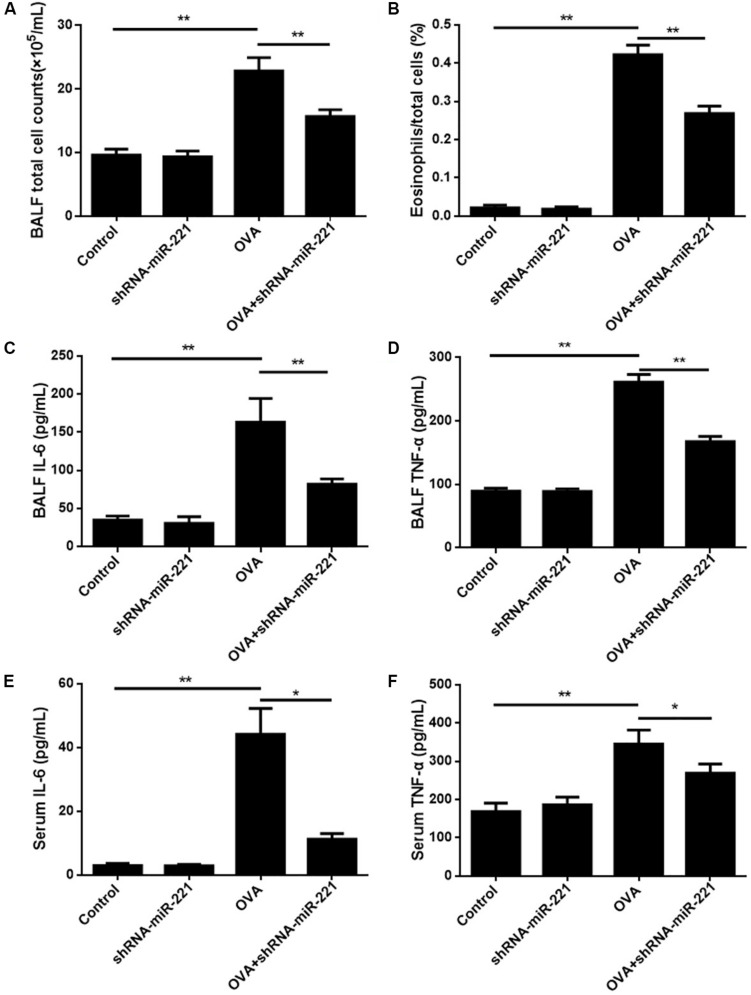
BALF inflammatory cell counts in different experimental groups and levels of cytokines in BALF and serum. **(A)** Total cell counts in BALF. **(B)** Ratios of eosinophil counts in BALF. **(C)** The level of IL-6 in BALF. **(D)** The level of TNF-α in BALF. **(E)** The level of IL-6 in serum. **(F)** The level of TNF-α in serum. Animal groups (in all panels): *n* = 6. **p < 0.01.

To examine the persistence of airway inflammation, we compared the levels of total cells, as well as the eosinophil percentages, in BALF. Figure 2A shows that the total count of inflammatory cells in the BALF was significantly higher (more than 2 times; *p* < 0.01) in mice that had undergone the conventional chronic protocol than in control mice. The levels of eosinophil percentages in the BALF were also elevated significantly in OVA-challenged mice compared with control mice (*p* < 0.01) (Figure 2B). The inhibition of miR-221 also significantly decreased the total cells and eosinophil percentages compared with the OVA group (*p* < 0.01) (Figures 2A,B). The cell count findings demonstrated that the downregulation of miR-221 by AAVs (which was significantly higher in the OVA group) effectively alleviated the OVA-exposure effect on elevated total cell counts and on the percentage of eosinophils in the BALF (Figures 2A,B) caused directly by OVA sensitization. The cytokines IL-6 and TNF-α in the BALF and serum were detected using ELISA kits to assess the nature of the cytokine response to OVA (Figure 2). IL-6 and TNF-α levels in the BALF and serum rose significantly in the OVA group compared to the control group (*p* < 0.05 or *p* < 0.01) (Figure 2). In addition, the inhibition of miR-221 resulted in a decline in IL-6 and TNF-α concentrations, showing a significant difference (*p* < 0.01) (Figure 2) in the OVA + shRNA-miR-221 group when compared to the OVA group.

### Effects of miR-221 Inhibition on Lung Histopathological Changes and on ECM Deposition

**FIGURE 3:**
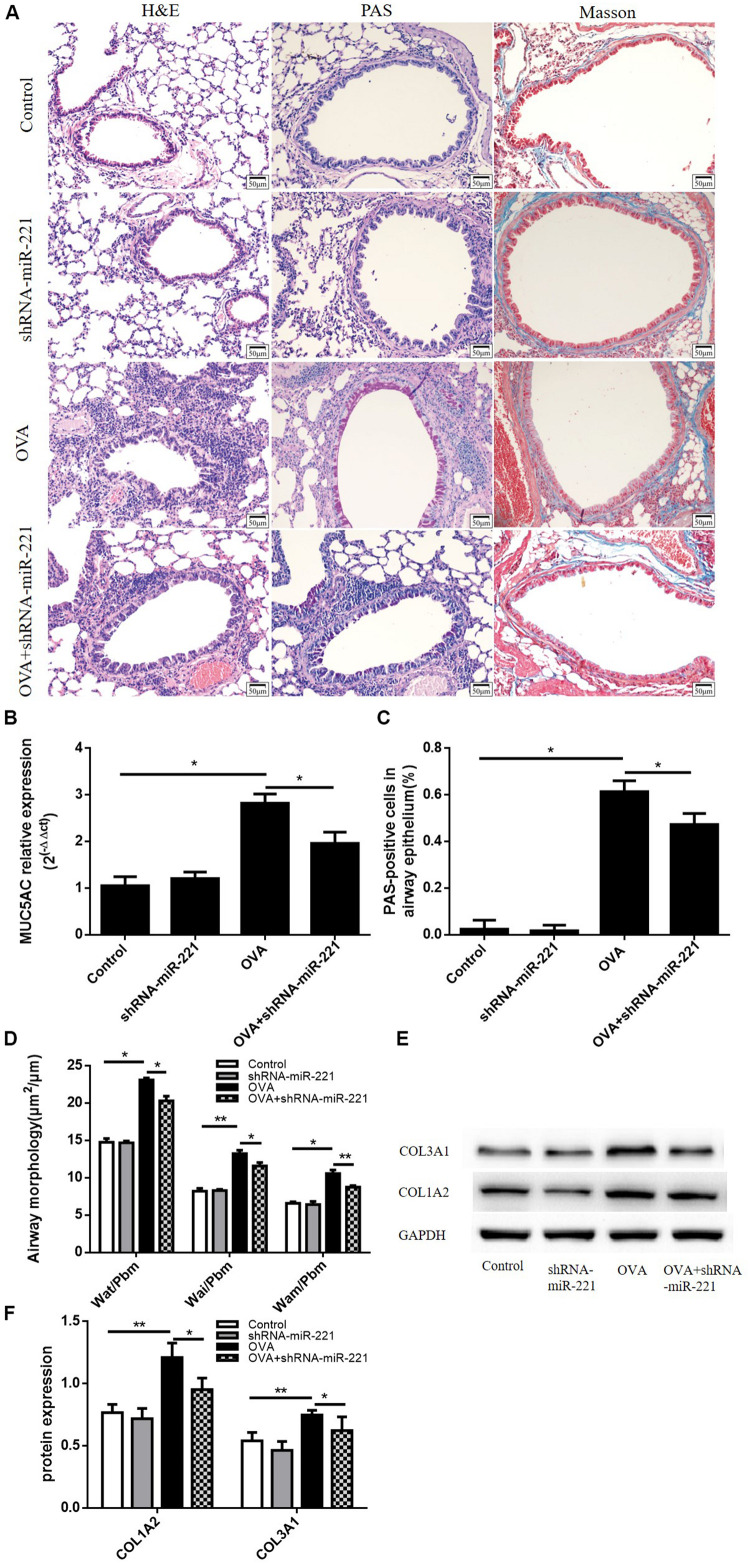
Representative lung sections from different staining methods and the expression levels of ECM proteins in the lung. **(A)** Hematoxylin and eosin (H&E) staining showing the infiltration of inflammatory cells, Periodic acid–Schiff (PAS) staining showing mucus cells (purple stain), Masson trichrome staining showing subepithelial collagen deposition (blue stain). **(B)** The mRNA expression of MUC5AC in lung tissue. **(C)** The percentage of PAS-positive epithelial cells. **(D)** Airway wall thickness was analyzed by HE-stained lung sections. Magnification, 200X; animal groups (in all panels): *n* = 6. *p < 0.05, **p < 0.01. **(E,F)** Representative western blots show the levels of type I collagen and type III collagen in the lungs. GAPDH was used as a loading control. Data are means ± SD from three experiments. *p < 0.05, **p < 0.01.

Airways from chronic asthmatic mice revealed severe peribronchial inflammatory infiltrate compared with control mice (Figure 3A). We measured the Wai, Pbm, Wat, and Wam of bronchi using Image Pro Plus 6.0 software. Airway walls appeared obviously thickened in asthmatic mice compared with control mice (Figure 3D). The inhibition of miR-221 could also significantly reduce goblet cell hyperplasia compared to the OVA group (Figure 3A). At the same time, the percentage of PAS-positive epithelial cells also significantly decreased in the OVA + shRNA-miR-221 group compared to the OVA group, consistent with the trend of the mRNA expression levels of MUC5AC in lung tissue (Figures 3B,C). Moreover, the downregulation of miR-221 attenuated airway collagen deposition in the lung tissue of mice (Figure 3A). Western blot analysis showed that compared to the control group, the expression of type I collagen and type III collagen was higher in the OVA group (Figures 3E,F), whereas miR-221 downregulation, induced by OVA, significantly decreased their expression. In summary, the inhibition of miR-221 could alleviate the collagen deposition in the ECM resulting from chronic asthma.

### Effects of PDGF-BB and EGF Stimulation on miR-221 Expression and ECM in ASMCs, and Effects of miR-221 Upregulation on the Deposition of the ECM in ASMCs

**FIGURE 4:**
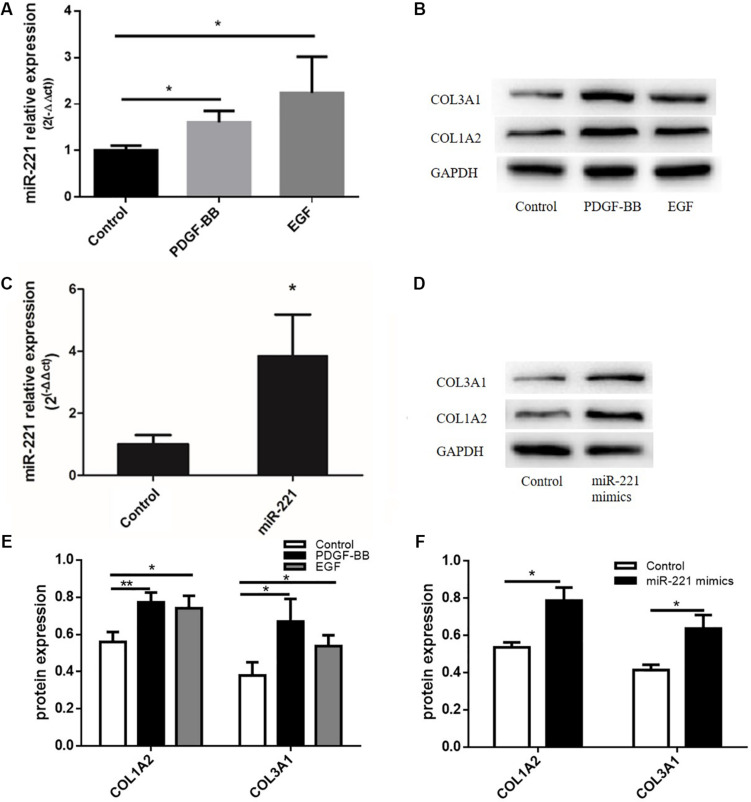
The protein expression levels of ECM after the stimulation of PDGF-BB and EGF in ASMCs and the protein expression levels of ECM after miR-221 overexpression in ASMCs. **(A)** The expression of miR-221 in ASMCs. **(B,E)** The expression of protein in response to the PDGF-BB and EGF stimulation. *p < 0.05. **(C)** The expression of miR-221 in ASMCs. **(D,F)** The expression of protein in response to miR-221 upregulation. Data were normalized to GAPDH expression and are presented as expression relative to controls. Data are means ± SD from three experiments. *p < 0.05, **p < 0.01.

Studies have shown that PDGF-BB can promote the expression of ECM proteins such as type I collagen and type III collagen. Meanwhile, EGF, a type of cytokine, plays an important role in the proliferation and differentiation of various cells. In our study, after transfection with miR-221 mimics, the expression of miR-221 was significantly higher than that of the control group (Figure 4C). With the overexpression of miR-221, the levels of COL1A2 and COL3A1 increased compared to the control group (Figures 4D,F). As shown in Figure 4A, the expression of miR-221 increased with the stimulation of PDGF-BB and EGF in ASMCs. PDGF-BB and EGF could induce the expression of type I collagen and type III collagen (Figures 4B,E). Thus, we found that miR-221 downregulation could inhibit airway inflammation and airway remodeling in asthmatic mice.

### Effects of miR-221 Upregulation on the Activity of the PI3K/AKT Signaling Pathway

**FIGURE 5:**
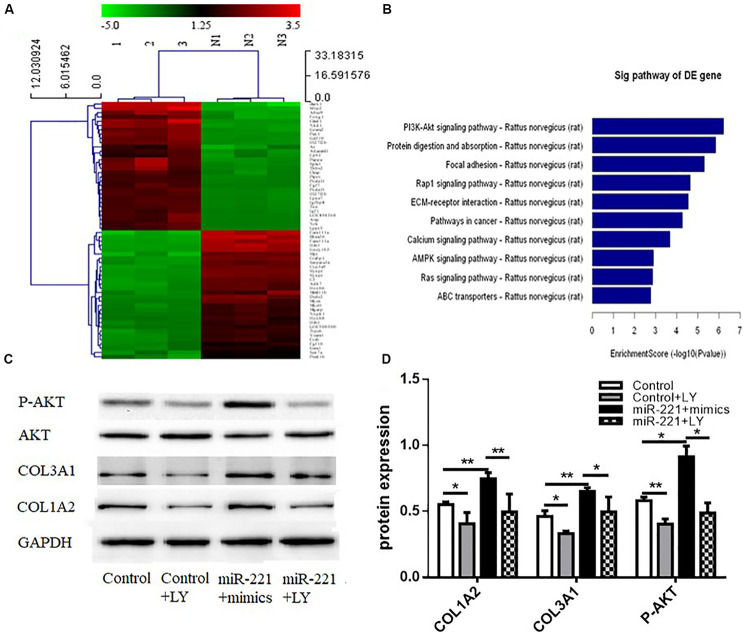
The activity of the PI3K/AKT signaling pathway in response to miR-221 upregulation. **(A)** The heat map of differential gene expression in different groups. **(B)** The changes in the signaling pathway. **(C,D)** The level of protein expression. Data were normalized to GAPDH expression and are presented as expression relative to controls. Data are means ± SD from three experiments. *p < 0.05, **p < 0.01.

A heat map of differential gene expression in the overexpression control group and the miR-221 overexpression group (miR-221 mimics) is shown in Figure 5A. miR-221 overexpression promoted the expression of the PI3K/AKT signaling pathway compared to the overexpressed control group (Figure 5B), suggesting that the PI3K/AKT signaling pathway might be involved in the expression of ECM proteins in ASMCs by miR-221. To further verify the role of the PI3K/AKT signaling pathway in the promotion of ECM expression in ASMCs by miR-221, we used LY294002, an inhibitor of the PI3K/AKT signaling pathway, to interfere with ASMCs. Western blot assays showed that the amount of total AKT protein was not significantly changed, but the protein level of phosphorylated (P-AKT) was significantly increased (*p* < 0.05 or *p* < 0.01) (Figures 5C,D). Compared with the miR-221 mimic group, the protein expression of COL1A2 and COL3A1 was decreased in the miR-221 mimics + LY294002 (PI3K/AKT inhibitor) group.

## Discussion

MicroRNAs have been implicated to have a fundamental role in asthma and in airway remodeling through the regulation of multiple signal transduction pathways involved in the pathogenesis of asthma. There is now increasing evidence that miRNAs (including miR-34/449, let-7, miR-19, miR-21, and miR-455) play potentially important roles in asthma. Simpson revealed that miR-19 is upregulated in asthma by promoting type 2 helper T cytokine production and amplifying inflammatory signaling (Solberg et al., 2012). miR-19a has been found to enhance airway epithelial proliferation as result of the loss of TGFBR2-mediated SMAD3 phosphorylation in ASMCs from patients with severe asthma. A previous study reported that miR-142 inhibits ASMC proliferation and promotes apoptosis during airway remodeling in asthmatic rats by inhibiting TGF-β expression via a mechanism involving the EGFR signaling pathway.

Meanwhile, Mayoral demonstrated that miR-221 can regulate the cell cycle of mast cells and also regulate the contraction and relaxation of ASMCs (Mayoral et al., 2011). The upregulation of miR-221 can also increase mast cell degranulation. Perry MM found that the inhibition of miR-221 can alleviate the proliferation of cells and the release of IL-6 in ASMCs from the asthmatic patients (Perry et al., 2014).

In our previous studies, we found that the expression of miR-221 was higher in asthmatic patients than in control subjects (Zhou et al., 2016). By qRT-PCR, we confirmed that the expression of miR-221 in the peripheral blood of asthmatic children was significantly higher than that of healthy children (Liu et al., 2012). Since miR-221 has been identified to be involved in the development of asthma, we explored whether and how miR-221 affected OVA-induced asthmatic mice in this study.

ASMCs are key players in airway pathologies such as augmented airway inflammation and airway remodeling. It has been recognized that abnormal ASMC proliferation, such as hypertrophy and hyperplasia, can result in the increased thickness of airway walls, contributing to the development of the airway remodeling observed in asthma (Céline et al., 2010; Boulet, 2018). Therefore, it is important to explore the underlying mechanism of ASMC proliferation. Plenty of studies have found that a variety of stimulating factors, including growth factors, hematogenic or inflammatory mediators, cytokines, and ECM proteins, can induce ASMC proliferation (Suojalehto et al., 2014). Growth factor-induced ASMC proliferation is considered the major cause of airway wall thickening in asthma, and increased levels of PDGF-BB have been noted, which contribute to ASMC proliferation (Liu et al., 2015; Dai et al., 2018). In this study as well, we demonstrated that the expression of miR-221 and the protein levels of ECM could be influenced by the simulation of PDGF-BB and EGF, inducing the proliferation of ASMCs. However, the underlying mechanism is not clear. Many types of inflammatory cells play roles in asthma by releasing inflammatory mediators that cause sustained chronic inflammation of the airways. This triggers bronchoconstriction and airway structural changes. We found that the inhibition of miR-221 caused higher inflammatory cell infiltration, goblet cell hyperplasia, mucus obstruction, and MUC5AC mRNA expression in lung tissue. Goblet cells are simple columnar epithelial cells that secrete gel-forming mucins, like the mucin MUC5AC (Kanoh et al., 2011; Ma et al., 2017). Studies have shown that goblet cell metaplasia and airway hypersecretion can occur in asthma (Chen et al., 2015).

The PI3K/AKT pathway is important for cell growth, differentiation, metabolism, survival, and apoptosis. Numerous studies have demonstrated that the PI3K/AKT pathway participates in the regulation of miRNAs, thereby affecting the proliferation of ASMCs (Liu Y et al., 2018; Wang et al., 2018). The PI3K/AKT pathway is involved in promoting ECM expression. The inhibition of miR-223 has been found to inhibit the deposition of ECM in ASMCs by targeting IGF-1R via the PI3K/AKT pathway (Wang Q. et al., 2015). Wang J reported that miR-29b could regulate the PI3K/AKT signaling pathway by negatively targeting PIK3R1 and AKT3, thereby reducing the expression of ECM proteins such as COL1A2 in mouse hepatic stellate cells (Wang J. et al., 2015). Osaki M also determined that the PI3K pathway could affect the proliferation of ASMCs in asthmatic patients and play an important role in the differentiation, activation, and production of cytokines of T cell receptors and T cells (Osaki et al., 2004). The above research indicates that the PI3K/AKT signaling pathway somehow participates in promoting ECM protein expression. This study also found that the expression of P-AKT in ASMCs increased after the overexpression of miR-221, which further suggests that miR-221 may promote the expression of ECM proteins in ASMCs by activating AKT and phosphorylating it.

Our findings shed new light by providing clues for the prevention and treatment of chronic asthma, especially airway remodeling. However, the limitations of this study should be mentioned. Since we have not identified the corresponding target gene of miR-221 yet (Supplementary Figure S1), further studies will need to focus on the exact mechanism of how miR-221 inhibition alleviates asthma.

## Conclusion

We propose that the downregulation of miR-221 may affect airway remodeling via the PI3K/AKT pathway in murine asthma. These findings highlight a novel aspect of the role of miR-221 in asthma and support the development of a novel therapeutic strategy based on this miRNA.

## Data Availability Statement

The raw data supporting the conclusions of this article will be made available by the authors, without undue reservation, to any qualified researcher.

## Ethics Statement

All animal experiments were approved by the local animal care committee of the National Defense Medical Center (approval number: IACUC-1702005).

## Author Contributions

FL, DZ, JP, and QY designed and performed the experiments, analyzed and interpreted the data, and wrote the manuscript. YZo, HD, and YZu performed the experiments. All authors contributed to the article and approved the submitted version.

## Conflict of Interest

The authors declare that the research was conducted in the absence of any commercial or financial relationships that could be construed as a potential conflict of interest.

## Supplementary Material

Many studies have confirmed that miR-221 can regulate the biological function of cells by targeting PTEN binding to activate PI3K. Du J showed that miR-221 could target PTEN to reduce the sensitivity of cervical cancer cells to gefitinib through the PI3K/AKT signaling pathway. Meanwhile, we demonstrated that the overexpression of miR-221 promoted the expression of the PI3K/AKT signaling pathway, suggesting that this pathway might be involved in the expression of ECM proteins in ASMCs through miR-221. This led us to wonder if PTEN could reduce airway remodeling by downregulating the expression of miR-221 via PI3K/AKT signaling. We show that the difference in the expression of PTEN with the inhibition of miR-221 was not significantly different between the groups.

FIGURE S1 **(A)** Protein expression levels of PTEN in an asthmatic mouse. GAPDH was used as a loading control. **(B)** Relative protein expression of PTEN. There was not significantly different between the inhibition of miR-221 compared to controls. We induced overexpression or inhibition of miR-221 in ASMCs by miR-221 mimics and miR-221 inhibitor.

FIGURE S2 The study’s experimental design and the experimental protocol to develop an OVA-induced mouse model of chronic asthma. BALB/c mice were sensitized by injecting a mixture of OVA with Imject Alum and then challenged with aerosolized OVA (concentration, 5%) for 8 weeks (thrice a week). The groups were as follows: (1) sensitization and challenge with saline along with AAV6-GFP infection (control group); (2) sensitization and challenge with saline along with AAV6-miR-221-sponge infection (shRNA-miR-221 group), (3) sensitization and challenge with OVA along with AAV6-GFP infection (OVA group), (4) sensitization and challenge with OVA along with AAV6-miR-221-sponge infection (OVA + shRNA-miR-221 group). We induced overexpression or inhibition of miR-221 in ASMCs by miR-221 mimics and miR-221 inhibitor. We investigated ASMC viability by using a CCK-8 assay. The results in Supplementary Figure S3 suggest that miR-221 could not affect ASMC viability.

FIGURE S3 The effect of miR-221 on ASMC viability. ASMCs were transfected with 50 nM miR-221 mimics and miR-221 inhibitor compared with control. Cell viability was then assessed 48 h post-transfection using a CCK-8 kit. Data are means ± SD from three independent experiments (*n* = 5).

## Abbreviations

AAV: adeno-associated virus
AHR: airway hyper-responsiveness
ASMCs: airway smooth muscle cells
BALF: Bronchoalveolar lavage fluid
ECM: extracellular matrix
EGF: epidermal growth factor
H&E: hematoxylin and eosin
i.p.: intraperitoneal
miR-221: microRNA-221
miRNAs: MicroRNAs
OVA: ovalbumin
PAS: periodic acid–Schiff stain
Pbm: airway basement membrane perimeter
PDGF-BB: platelet-derived growth factor
Re: expiratory resistance
Ri: inspiratory resistance
UTR: untranslated region
Wai: airway wall thickness
Wam: airway smooth muscle area
Wat: airway wall area.
