# Supplementary figures and images for: MicroRNA-221 Modulates Airway Remodeling via the PI3K/AKT Pathway in OVA-Induced Chronic Murine Asthma
Source: Front Cell Dev Biol. 2020 Jun 30;8:495. doi: 10.3389/fcell.2020.00495 (PMC7344209; doi:10.3389/fcell.2020.00495)

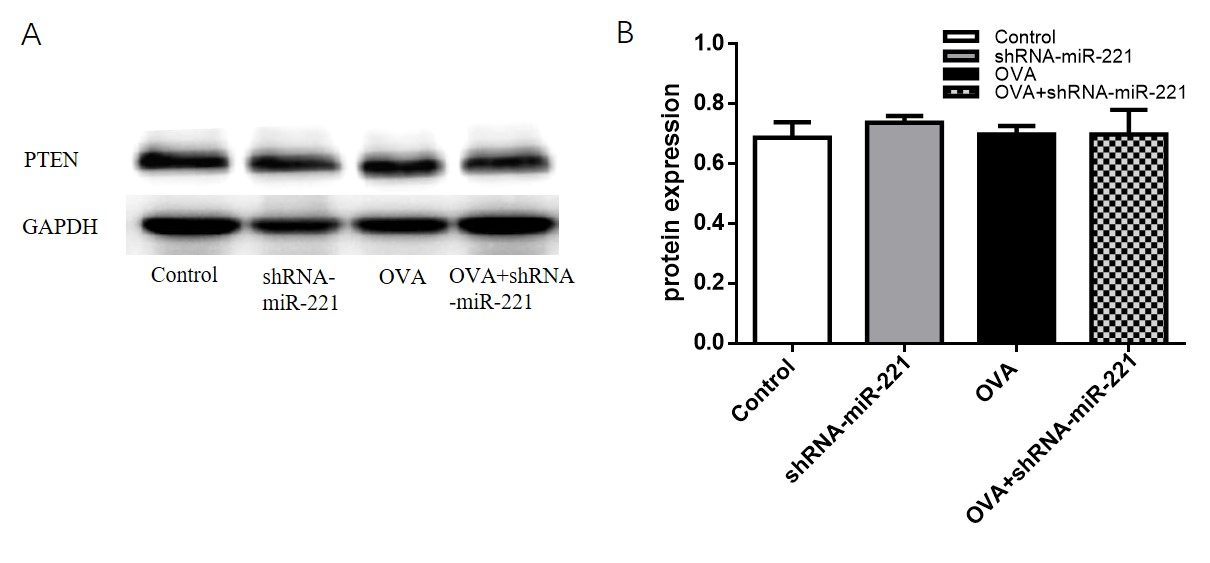

Supplement: FIGURE S1 — (A) Protein expression levels of PTEN in an asthmatic mouse. GAPDH was used as a loading control. (B) Relative protein expression of PTEN. There was not significantly different between the inhibition of miR-221 compared to controls. We induced overexpression or inhibition of miR-221 in ASMCs by miR-221 mimics and miR-221 inhibitor. [file Image_1.JPEG]

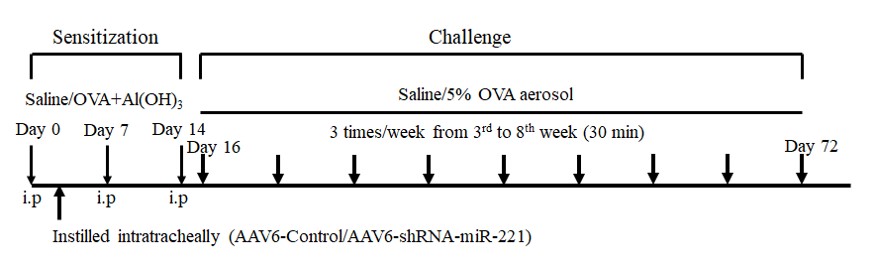

Supplement: FIGURE S2 — The study’s experimental design and the experimental protocol to develop an OVA-induced mouse model of chronic asthma. BALB/c mice were sensitized by injecting a mixture of OVA with Imject Alum and then challenged with aerosolized OVA (concentration, 5%) for 8 weeks (thrice a week). The groups were as follows: (1) sensitization and challenge with saline along with AAV6-GFP infection (control group); (2) sensitization and challenge with saline along with AAV6-miR-221-sponge infection (shRNA-miR-221 group), (3) sensitization and challenge with OVA along with AAV6-GFP infection (OVA group), (4) sensitization and challenge with OVA along with AAV6-miR-221-sponge infection (OVA + shRNA-miR-221 group). We induced overexpression or inhibition of miR-221 in ASMCs by miR-221 mimics and miR-221 inhibitor. We investigated ASMC viability by using a CCK-8 assay. The results in Supplementary Figure S3 suggest that miR-221 could not affect ASMC viability. [file Image_2.JPEG]

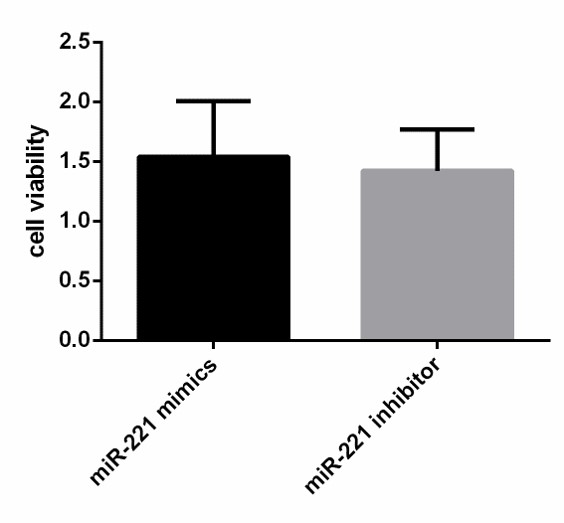

Supplement: FIGURE S3 — The effect of miR-221 on ASMC viability. ASMCs were transfected with 50 nM miR-221 mimics and miR-221 inhibitor compared with control. Cell viability was then assessed 48 h post-transfection using a CCK-8 kit. Data are means ± SD from three independent experiments (n = 5). [file Image_3.JPEG]
